# Medical students’ feedback regarding their clinical learning environment in primary healthcare: a qualitative study

**DOI:** 10.1186/s12909-016-0837-4

**Published:** 2016-12-13

**Authors:** Helena Salminen, Eva Öhman, Terese Stenfors-Hayes

**Affiliations:** 1 Division of Family Medicine, Department of Neurobiology, Care Sciences and Society, Karolinska Institutet, Alfred Nobels Allé 23, 141 83 Huddinge, Sweden; 2 Department of Learning, Informatics, Management and Ethics (LIME), Karolinska Institutet, Solna, Sweden

**Keywords:** Clinical learning environment, Students’ perceptions, Qualitative study, Primary healthcare

## Abstract

**Background:**

An increasing part of medical students’ learning takes place in primary healthcare (PHC) but little is known about how the students perceive PHC as a clinical learning environment. This study aimed to explore medical students’ perceptions of the clinical learning environment in PHC and how these vary with stage of education.

**Methods:**

Free-text course evaluation comments from students in nine different semesters during spring 2014 were analysed using qualitative content analysis. The students had placements in PHC from the first semester, progressing through the whole 5.5 year medical programme, and this was their main clinical training environment during the final 11^th^ semester.

**Results:**

In total, 800 students (56%) agreed to participate in the study and 437 of these (54%) provided comments. Two overall themes were identified: the supervisor was the central factor that determined the meaningfulness of the placement at all stages of the education, and basic prerequisites for perceived clinical learning were to have an active role in an authentic clinical context and to be trusted to work independently with patients.

The three main categories found under these themes were: i) the perceived relationship with the supervisor; ii) the perceived journey to become a doctor; and iii) the perceived structure and culture.

**Conclusion:**

The supervisor’s role was perceived as central at all stages of the education but the focus changed for other aspects, related to the students’ professional development. The need for trust and independence in patient work increased towards the end of the education.

## Background

The trend today is that an increasing share of medical students’ learning takes place in primary healthcare (PHC) [1]. This is the natural result of the changes in many of our healthcare systems, where most patients are taken care of in healthcare units outside the hospitals, which makes common conditions rare at our hospitals. In all Scandinavian countries, for example, the trend is that an increasing proportion of all medical conditions are handled in PHC. Few studies have addressed medical students’ perceptions of their clinical learning environment in PHC as it is a relatively new learning environment, albeit of growing importance [2–5]. In this study we explore how medical students perceive this learning environment at different stages of their education.

Learning in clinical environments is complex and has its special challenges. Transformative learning [6] gives a comprehensive theory to understand the kind of learning that takes places in clinical settings. During clinical practice the students construct new knowledge in a setting that is relevant and meaningful for them, where they will act as professionals in future. This clinical setting builds frames of reference in social interaction and makes their learning coherent [6, 7]. During the process their frames of reference change and the students get new perspectives that involve learning clinical and diagnostic skills, communicating with patients, peers, supervisors and other healthcare professionals, and acting in their profession. It is common in Sweden to use PHC for vertical integration of learning through all stages of the medical education, giving the students a PHC perspective on everything they learn in other contexts. Ideally, their understanding of the role of PHC and their confidence as future physicians is transformed during the process.

Many aspects are important for the learning environment and influence the students’ learning. It has been shown that PHC offers students a variety of patient contact, which is appreciated by the students and perceived as stimulating and meaningful to their learning [2], and worldwide, many different ways of training students in this environment exist [3]. When the conditions for students are optimal, they have the chance during their clinical placements to transfer theory to practice and to develop their professional identities [2]. Furthermore, teachers, patients, specialists and the general population may also benefit from successful clinical placements in PHC [4]. For a satisfactory clinical learning environment, the most important aspect found in previous studies is the relationship with the supervisor [8, 9]. There are also other aspects in the quality of the physical environment, such as the attitudes of other staff members, and practical matters, such as access to computers, that contribute to the way students perceive their clinical learning environment [10–12]. The importance of the relationship with the supervisor may be even more important in PHC than elsewhere in clinical settings, as the whole structure of PHC is centred on the individual meetings between the doctor and the patient. This makes the supervising doctor a key person who gives the student access to the patients. This relationship may be more important than the methods used in supervision [13, 14]. The trust in the relationship with the supervisor also affects the feedback the student gets and how confident the student feels in the situation. These feelings and the relationship are important for the student’s learning process in the clinical context [6]. All this makes it essential to have in-depth knowledge of how the students experience PHC as a learning environment. However, our hypothesis is that students’ needs and perceptions vary depending on what stage of their education they are in. To our knowledge, no previous studies exist exploring this perspective using a qualitative explorative methodology.

The aim of this study was to explore medical students’ perceptions of the clinical learning environment in primary healthcare and how these vary at different stages of their education.

## Methods

### Study design

A qualitative study based on content analysis [15] of medical students’ free-text comments in the student experience survey of the clinical learning environment.

### Context

Undergraduate medical education is 5.5 years long in Sweden. The education leads to a medical degree as a doctor but doctors are not licensed until they have completed an 18-month internship. PHC was introduced as a strand in the study programme in medicine at the medical school in our study (Karolinska Institutet, Sweden) in 2007. Placements in PHC build a thread that leads to horizontal and vertical integration in the programme. Medical students have clinical placements in PHC for 10 weeks, spread over nine semesters. The clinical period per semester at the PHC centre varies from 4 to 7 days. PHC is both the first clinical contact during first semester and the last training environment during the final semester before students graduate. The role of the supervisor should differ depending on which stage the student is at, and the supervisors are instructed to give the students a lot of independence and autonomy under safe supervision, towards the end of their education. The primary care part of the medical programme has its own learning goals for each semester. There is a clear progression in these goals. The learning goals reflect both the learning goals for Family Medicine and the courses or themes where students have placements in PHC. Primary care is also responsible for the training in basic consultation skills. During the first semesters, the students mainly train with different methods, such as videotaped consultations, to understand the patient’s agenda (the patient’s ideas, concerns and expectations); from semester four on, history taking is added to the training; and during the last semester students also learn to involve and motivate the patient and build a shared understanding with the patient. During the last semester, students are assessed on an entire filmed patient encounter from the student’s practice in PHC.

### Population and data collection

The population in this study were medical students from one Swedish medical school from semesters 1–11, with the exception of semesters 7 and 10 (these semesters did not contain any placement in PHC). A questionnaire assessing the medical students’ experience of the clinical learning environment in PHC was sent electronically to all 1246 medical students in spring semester 2014. There were around 120–150 students per semester, distributed at around 200 PHC centres around Stockholm.

### The instrument clinical learning and supervision

The instrument Clinical Learning and Supervision (CLES) was initially created for evaluation of the clinical learning environment of nursing students [16]. We have adapted and validated it for medical students in PHC, and as part of this process we have added two open-ended questions: what the students thought functioned well at the PHC centre and what could be improved. These free-text answers were the data analysed in this study.

### Analysis of the data

We conducted a qualitative content analysis of the students’ free-text answers and analysed the manifest and latent content by first building meaning units, then categorizing the units and finally creating themes [15]. The categories and sub-categories were based on the manifest content of the data, while the themes reflected more the latent content of the data.

First, the data were read several times by all the researchers and discussed. The first author built meaning units and the sub-categories and categories were then discussed with the other two researchers until consensus was reached. Main themes were identified and discussed. Special effort was made to identify longitudinal changes in students’ perceptions through their education. Every category found was analysed from that perspective and the comments were reread several times during the process. The researchers had different healthcare backgrounds and different experiences of medical education. The first author’s role as responsible for the PHC part of the medical students’ education was considered, but as she rarely has direct contact with the students, the risk that the students would not be honest in their comments was considered minimal.

### Ethical considerations

Ethical approval was obtained for the study from the Regional Ethical Review Board in Stockholm. Each student ticked a box in the evaluation instrument, in order to give consent to take part in the study. Information about the study was sent together with the instrument by email to the students. All data were anonymous and it was not possible to link students’ identities to their responses.

## Results

In total, 800 medical students (56% of a total of 1246 students) from nine semesters agreed to participate in the study and 437 of these (54%) provided comments.

Two overall themes were identified. These two central themes permeated the data and could be found permeating the contents in many of the categories.

The first overall theme found was that ***the supervisor was the central factor that determined the meaningfulness of the placement at all stages of the education*** **.**

The perceived meaningfulness of a placement depended on how the relation with the supervisor was viewed. At every stage of their education, students commented on the feedback they received from the supervisor. At the early stages they wanted their supervisor to explain what they did and how they reasoned in relation to the patient encounters. Towards the end of their education, the students wanted feedback that was tailored to where they were in their development, and to be trusted to handle patients independently.

*“A placement in PHC depends 100% on the quality of the supervisor, I had a good supervisor this week. The same PHC centre with another supervisor could have been a bad week…”* (682, Semester 8 student)

The second overall theme was that ***basic prerequisites for perceived clinical learning were to have an active role in an authentic clinical context and to be trusted to work independently with patients.***

The students highly appreciated the opportunity to meet real patients early in their education. Their comments reflected their perceived journey to become a doctor. Training communication with patients and clinical skills was in focus at all stages while their need for autonomy increased towards the end of their education. Table 1 shows the main themes and categories of the study. The themes express the latent content while the manifest content was expressed in three categories:

**Table 1:** The main themes and categories of the study

Main themes	Categories
	The perceived relationship with the supervisor
The supervisor was the central factor that determined the meaningfulness of the placement at all stages of the education	
	The perceived journey to become a doctor
Basic prerequisites for perceived clinical learning were to have an active role in an authentic clinical context and to be trusted to work independently with patients	
	The perceived structure and culture

1. The perceived relationship with the supervisor
2. The perceived journey to become a doctor
3. The perceived structure and culture

### The perceived relationship with the supervisor

The relationship with the supervisor was found to be the most central determinant of the students’ perceived satisfaction with their placement. The perceived trust was already important right from the beginning. To let the student measure the blood pressure of the patient, for example, was to show trust at an early stage where the student’s competence to handle more complicated tasks was limited.

*“Then I got the opportunity to practise some practical skills such as listening to the lungs, measuring blood pressure and checking some ears, which was very good.”* (446, Semester 1 student)

At early stages the students wanted feedback on how their supervisor would handle the patient, while at the end of the education the students wanted their supervisor to give feedback on how they reasoned and did not just want the supervisor to tell them about how he or she reasoned.

*“After every patient encounter, I could go and fetch my supervisor and summarize the patient case, discuss differential diagnoses and give suggestions for further steps and investigations.”* (803, Semester 9 student)

During the early terms, many students perceived the supervisor as a role model in patient encounters. They appreciated the possibility to gain some early insights into their future profession. They also appreciated seeing different ways a doctor can act in communication with patients.

The continuity of the supervision during the placement was considered important by the students at all stages. Most students preferred to have one supervisor who kept track of what they did at the PHC centre. If many doctors shared the task of supervising, there was a perceived risk that nobody would get a whole picture of how the student was doing, nobody would know their learning goals and they would not adjust the supervision to the stage of education the student was in.

### The perceived journey to become a doctor

Having early patient contacts and seeing real doctors in action were greatly appreciated by the students during the first semesters. They described it as getting anchored in reality and gaining some insight into what a real working day might look like for a doctor.

*“Very stimulating to see how a real working day can be and to get some foundation in the reality.”* (444, Semester 1 student)

The students got their introductory course in medical diagnostic skills in semester four but they were introduced to simpler clinical skills in semester one when they had PHC. They also practised communication skills with recorded consultations with patients from semester two at their PHC centre. This early introduction to process skills during the first three semesters was something the students often appreciated in their comments.

At all stages, the students expressed awareness of whether their placement at the PHC centre matched the learning goals or not. During the first semesters they wanted to do everything on their checklists for the day at the PHC centre, later on from semester five (the course in Clinical Medicine), their learning goals were not fulfilled if they could not meet enough patients of a selected kind, such as patients with minor surgical problems, during the course in clinical surgery. From semester five, they entered the real clinical stages of the programme and were expected to train on real patients using their newly acquired skills. The students seemed to be eager to train only on patients that were suitable for the learning goals of the course; patients with unselected medical problems were perceived as not necessary for the learning. The demand for patients as examples of their specific conditions was frequent in semesters five, six and eight, the courses in clinical medicine and clinical surgery. Especially during the course in surgery the students wanted the PHC centres to select suitable patients for them, preferably patients suitable for minor surgery, such as removing birth marks.

*“A big minus was that I did not have that many surgical patients, most of them had medical problems, no training in minor surgery, which was what I had looked forward to.”* (684, Semester 8 student)

A turning point in the students’ comments from wanting selected to unselected patients to handle was observed during semesters 9 and 11. During the last semester they wanted to meet unselected patients in order to prepare for their internship and their station-based examination before they graduated. It became increasingly important in the later semesters for the students to have autonomy to work more and more independently. Given this autonomy, they still wanted it to be a safe environment with constructive feedback from their supervisors but with a high degree of independence. The number of comments on how important autonomy was for the students’ learning experience was very large. The need to handle patients of their own became apparent in the students’ comments from semester five and increased in the number of comments towards their graduation.

### The perceived structure and culture

If the whole staff was involved in taking care of the students, the students’ satisfaction with their placement seemed higher. When all staff members were perceived to be engaged in taking care of the students, they felt that they belonged to and were a part of the workplace. Even if they had a fantastic supervisor, this person alone could not create a good atmosphere at PHC centre around supervising students. This perceived good atmosphere required the engagement of all staff.

*“As soon as the receptionists saw us, they beamed and were welcoming and they always helped us.”* (499, Semester 2 student)

The most common complaint was that the PHC centre had not prepared for the students’ placement. Sometimes they had even missed that the students were coming. If the main supervisor was absent or sick, there might not be any backup plan. Sometimes no information was sent to students before the placement. Quite often the rest of the staff had no idea why the students were there, and other doctors who were not responsible as supervisors had a very limited interest in taking care of the students.

*“Better communication is desirable between the supervisor who is responsible and the other doctors so that they know what is required.”* (492, Semester 2 student)

Students thought that the doctors who took good care of their students also took good care of their patients. The students noticed that their supervisors and other staff often seemed to work under stress. Occasionally the students commented on the short time the patients had for their encounters with the doctor. If they perceived dysfunctional supervision, they often also perceived abnormalities in how patients were treated.

*“When I was there with her (the supervisor) during the patient meetings, we students just had to sit there and watch. On several occasions she was unkind to patients and did not listen to them.”* (493, Semester 2 student)

*“The doctor I followed most of the time, was so superb! She had always time for my questions… and was also so professional! And had a great way of handling the patients. She made me look forward to going to the PHC centre!”* (540, Semester 3 student)

Sometimes the logistics just did not work and there were gaps in the schedule of students when the staff had meetings. This irritated the students who had often travelled a long way to get to the PHC centre and sometimes felt the gaps were a waste of valuable time. The long travelling distances in the Stockholm region also contributed to the logistical problems perceived by the students.

The students commented on things that had not worked or had worked well in their physical learning environment. Access to computers and log-ins to the electronic records caused occasional problems, and many PHC centres could not offer separate rooms for students. The number of comments about the structure of the placements and the perceived culture at the PHC centre seemed to increase somewhat from semester five, the stage where the students were expected to work more independently with patients. The demands made of the physical learning environment reflected the students’ need for increasing autonomy towards the end of their education, with an increasing number of comments about having one’s own patients, own office, own access to the medical records, all tools for independence that facilitate acting in the role of a professional.

*“The environment allowed me to grow in the role of a doctor – my own computer, own bookings, a real office to examine the patient in.”* (701, Semester 8 student)

## Discussion

The main findings of this study were that medical students in PHC viewed the relationship with their supervisor as the central determinant for the outcome of a clinical placement in PHC and that the prerequisite for them to perceive that they learned something and that it was meaningful was that they could have an active role and be accepted participants, trusted to take care of patients independently. These findings are in concordance with other studies where the focus has been on the various dimensions that influence students’ learning in a clinical context [17–19]. The relationship to the supervisor has previously been shown to be of importance both at hospitals and in primary care [8, 9, 16].

What this study adds is a perspective on how medical students perceive different stages throughout the whole medical programme and how students’ feedback changes focus at different stages of their education, reflecting the students’ needs in their professional development. Their comments reflect their progression towards their future profession as doctors. The relationship with the supervisor was considered a critical determinant of the outcome of a placement at all stages of the medical students’ education, while other aspects varied in importance throughout the education. Trust and independence in patient work increased in perceived importance towards the end of the education.

During their learning process throughout the medical programme, students’ frames of reference change and they get new perspectives that involve learning clinical and diagnostic skills but also communicating with patients, peers, supervisors and other healthcare professionals and acting in their profession. This change of reference frames becomes visible during a thread such as PHC where students return every semester and continuously integrate new knowledge and skills in the context of PHC, differing from what they learn in hospitals. In PHC the perspective on the patient is broadened; the patient’s family, social context and neighbourhood must be taken into account. This transforms the student’s perspectives during their journey to become a doctor. The comments from students reflect needs in their learning process and how these needs change. Initially, the students lack a frame of reference; they need a lot of support from a supervisor and react to how this role model behaves. Later, the students need to try the new role of a doctor and build their self-confidence through successful patient encounters and other experiences at the workplace. They require an increasing amount of independence and trust towards the end of their education but are still dependent on guidance. This is in concordance with the supportive trust that Dornan and colleagues found important for professional development [19]. While the prerequisite for learning at all stages seemed to be a good relationship with the supervisor, the requirement of having one’s own patients, own bookings, own office and independent acting in the profession increased towards the end of the education, near their graduation. Students not only need to acquire knowledge and skills but also to feel confident in acting in their profession [19]. Our study also shows how the students felt that they grew when the supervisors showed trust in them.

Active student participation has been shown to be a key construct of the clinical learning environment [20]. Active participation in work with patients is also crucial for the emotional development of medical students [21]. Moreover, in our study, active participation in an authentic context was a key theme in the students’ perceptions. Manninen and colleagues found that students experienced both external authenticity (just being in a real workplace with real patients) and internal authenticity when they had responsibility under guidance and support [18]. This is equivalent to how the students in our study perceived their placement as meaningful when they were given enough but not too much support and independence to handle patients to their level of competence. The importance of authenticity relates well to the theory of communities of practice [22], because when students perceive both external and internal authenticity they are on their way to becoming more central participants in the community of practice of the workplace. The more engaged and more central participants the students become in the team in the authentic clinical setting where they have a meaningful role in the team of the community of practice in handling real patient encounters, and the nearer they are to their role in their future profession as doctors, the more authentic their learning experiences become.

Early patient contact has been shown to be very useful to motivate students in their more preclinical years as it triggers the students’ professional development and anchors their more factual knowledge [23]. These findings were also supported by our study. During the third and fourth years the students seemed to focus on learning to diagnose different diseases and wanted the PHC centres to select appropriate patients. Towards the end of their education they asked for unselected patients and wanted to handle the patients independently.

The findings in our study are well aligned with the theory of transformative learning [6]. When the learning environment is optimal, they perceive, according to their comments, support from their supervisors but are allowed to act independently to the limit of their competence and experience meaningful learning in relation to their future profession. They experience the transformation from a student to doctor, and they not only learn new things but are themselves transformed in the preparation for their future role as doctors. Their comments in our study reflect their needs during that process.

Regarding the credibility of the present study, all medical students at all stages of the medical programme with placements in PHC were invited to participate in the study. Each semester had 120–150 students, so the number of comments in the study, with the majority of the students willing to participate and all free-text answers from those students included, represented a rich material. Not all students provided free-text answers and we do not know for certain how these students might differ from those who did so. Our experience, based on using these same open-ended questions during the past 5 years, is that the comments used in this study are in concordance with the comments we get every semester. Further research may include in-depth interviews with students as well as comparisons with students’ experiences from other universities.

The results of this study have been included in faculty development of clinical supervisors. Students demand increasing autonomy the more advanced they are in their knowledge and skills, so the challenge is to adapt the supervision both to the student’s stage in education and to the student’s individual needs.

## Conclusions

Medical students’ perceptions of the meaningfulness of their placements in PHC depend mainly on the quality of their relation to their supervisor, but the focus changes for other aspects during the education, related to the stages in the students’ professional development. To have an active role in an authentic context was felt to be important for their professional development. The perceived need for trust and independence increased towards the end of their education.

## Abbreviations

CLES: Clinical learning and supervision instrument
PHC: Primary healthcare
